# Multi-Scale Modeling in Forming Limits Analysis of SUS430/Al1050/TA1 Laminates: Integrating Crystal Plasticity Finite Element with M–K Theory

**DOI:** 10.3390/ma19020390

**Published:** 2026-01-18

**Authors:** Xin Li, Chunguo Liu, Yunfeng Bai

**Affiliations:** 1 College of Materials Science and Engineering, Jilin University, Changchun 130025, China; xin_l20@mails.jlu.edu.cn (X.L.); baiyf24@mails.jlu.edu.cn (Y.B.); 2 Roll Forging Research Institute, Jilin University, Changchun 130025, China

**Keywords:** crystal plasticity finite element, forming limit diagram, M–K model, laminated metal composites

## Abstract

Numerical simulations of the forming limit diagram (FLD) for SUS430/Al1050/TA1 laminated metal composites (LMCs) are conducted through the crystal plasticity finite element (CPFE) model integrated with the Marciniak–Kuczyński (M–K) theory. Representative volume elements (RVEs) that reconstruct the measured crystallographic texture, as characterized by electron backscatter diffraction (EBSD), are developed. The optimal grain number and mesh density for the RVE are calibrated through convergence analysis by curve-fitting simulated stress–strain responses to the uniaxial tensile data. The established multi-scale model successfully predicts the FLDs of the SUS430/Al1050/TA1 laminated sheet under two stacking sequences, namely, the SUS layer or the TA1 layer in contact with the die. The Nakazima test results validate the effectiveness of the proposed model as an efficient and accurate predictive tool. This study extends the CPFE–MK framework to multi-layer LMCs, overcoming the limitations of conventional single-layer models, which incorporate FCC, BCC, and HCP crystalline structures. Furthermore, the deformation-induced texture evolution under different loading paths is analyzed, establishing the relationship between micro-scale deformation mechanisms and the macro-scale forming behavior.

## 1. Introduction

In recent years, laminated metal composites (LMCs) have been attracting much attention in aerospace, automotive, domestic parts, and various industrial applications, due to the advantages of desirable mechanical properties, favorable corrosion resistance, and cost-effectiveness. LMCs can achieve superior performance by balancing the properties of two or more heterogeneous metal layers compared to constituent materials [1,2,3]. However, their forming behavior differs from that of monolithic metals. LMC exhibits complex deformation mechanisms, including interlayer interactions, strain partitioning among different layers, and damage initiation and evolution, leading to challenges in constitutive modeling [4,5,6].

The forming limit diagram (FLD) is the primary tool used to assess the plastic deformation ability in sheet metal-forming analysis. Traditionally, FLDs were determined through experimental methods such as Nakazima tests, and strain distributions were measured using digital image correlation (DIC). While these experiments provide critical data, obtaining the FLD through measurement is typically time-consuming and costly, which has prompted many researchers to develop various theoretical models to predict forming limits. Since the introduction of the M–K model by Marciniak and Kuczyński, FLDs of sheet metals under various stress states have been widely predicted through this computational approach [7]. The core of the M–K theory is the assumption that local initial imperfection exists within the material, which serves as the nucleation site for necking during deformation. While numerous studies have combined the M–K model with phenomenological constitutive models that characterize the shape and evolution of yield surfaces, the application of this combined theoretical framework in multi-layer metal sheets remains limited [8,9,10]. On the one hand, the accuracy of the M–K method heavily relies on the input constitutive laws, which are typically derived from simplified assumptions, ignoring the complexity of the multi-layer structure [11]. On the other hand, the performance of the laminates is affected by various factors, such as the properties of the constituent metals, the stacking sequence, and the interface strength. Therefore, many constitutive parameters are involved in the model, often requiring extensive mechanical testing for parameter calibration. Hashemi and Karajibani utilized Barlat and Lian’s 1989 anisotropic yield function combined with the M–K model to predict the FLDs of the Al–Cu laminated sheet. The model involved four nonlinear equations that needed to be solved simultaneously [12].

Crystal plasticity theory is an appropriate tool for understanding the micromechanisms of the plastic response. Crystal plasticity modeling can be categorized in terms of the homogenization scheme applied. The well known viscoplastic self-consistent (VPSC) model represents a typical method. Furthermore, crystal plasticity can also be incorporated in combination with a finite element model, where the compatibility and force equilibrium can be enforced from a finite element perspective. Crystal plasticity finite element (CPFE) incorporates the microstructural characteristics of the material into the constitutive framework, deriving the macroscopic plastic mechanical properties of the sheet metals from the microscopic responses of the grains by satisfying the compatibility and force equilibrium within the M–K model [13,14]. This synergistic approach, known as the CPFE–MK model, enhances the accuracy of formability predictions by employing the CPFE method to capture micro-scale deformation mechanisms while utilizing the M–K model to analyze macro-scale instability evolution. Previous studies based on the CPFE–MK method have been predominantly confined to single-layer metals, where microstructural homogeneity facilitates model implementation [15,16,17,18]. The size optimization of the CPFE model can significantly improve computational efficiency while maintaining the prediction accuracy of sheet metal formability [19]. Amelirad et al. determined the minimal grain count through thickness, minimal elements per grain, and minimal total grain count required for convergence of the stress–strain curve through a series of simulations [20]. Tran et al. also studied the size effect on the forming limit curves of the ultra-thin ferritic stainless-steel sheet using the CPFE–MK method [21]. In addition to the size effects, initial texture equally governs formability predictions. Wu et al. demonstrated that localized plastic instability depended critically on the initial texture and grain orientation distribution [22]. Azhari et al. constructed CPFE models using two distinct approaches: a series of parallel EBSD images obtained from sectioned annealed L-PBF Ti-6Al-4V alloys, and three orthogonal EBSD images [23]. Both methods successfully captured the actual polycrystalline microstructure with comparable prediction accuracy. During the deformation process, the microstructure of the polycrystalline material undergoes a notable transformation, and the evolution of the crystal texture also has a significant effect on the FLD and mechanical behavior of the materials. The crystal plasticity theory combined with M–K analysis is being widely used to assess the sheet metal formability. This framework facilitates the evaluation of key parameter effects, including material strain rates, slip systems, initial imperfection factor, and loading paths on FLDs predictions [24,25,26,27]. The studies mentioned above have substantially advanced FLD modeling comprehension; however, these studies mainly focus on monolithic metals, and the M–K model based on the CPFE method has not yet been established for LMC sheets.

The present study proposed a multi-scale framework applicable to well bonded LMC sheets for the first time based on CPFE incorporated within the M–K method. SUS430/Al1050/TA1 laminate was used as the study material, containing FCC, BCC, and HCP crystal structures with wide applicability. The crystal textures of all the constituent materials were characterized by EBSD. A MATLAB script was developed that can import the measured EBSD data and crystal constitutive parameters to create a three-dimensional (3D) representative volume element (RVE). Concerning the computational efficiency and costs, the optimal total grain count and element number in each grain required to ensure the accuracy of the predicted stress–strain curves were determined through convergence analysis. RVEs with optimal size were able to effectively reproduce stress–strain responses during uniaxial tension tests. The modified CPFE–MK model was used to simulate the FLDs of the SUS430/Al1050/TA1 sheet. The forming limits of two stacking sequences, with either the steel or Ti layer in contact with the die, were successfully predicted when validated against the Nakashima test results. In addition, the deformation-induced textures under various loading paths were comprehensively analyzed to account for the effect of the microstructure at the microscopic scale on the macro-scale properties.

## 2. Materials and Methods

### 2.1. Initial Texture

**Figure 1:**
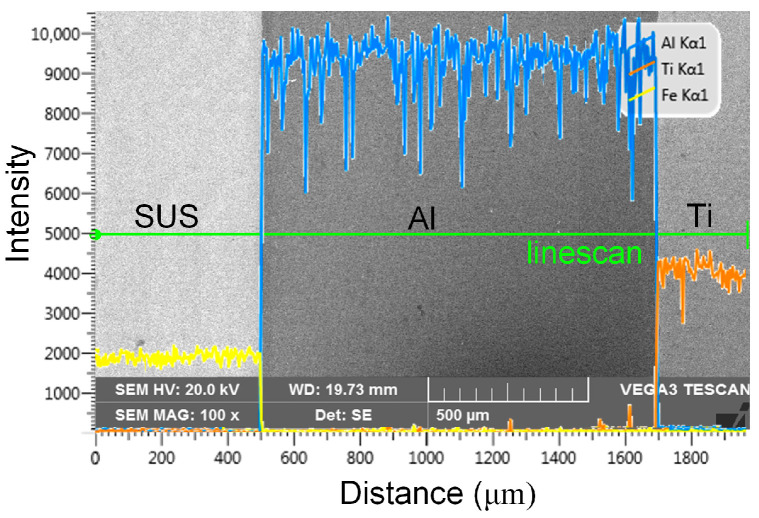
The SEM figure of the SUS430/Al1050/TA1 LMC with element distribution lines (yellow: Fe; orange: Ti; blue: Al).

**Figure 2:**
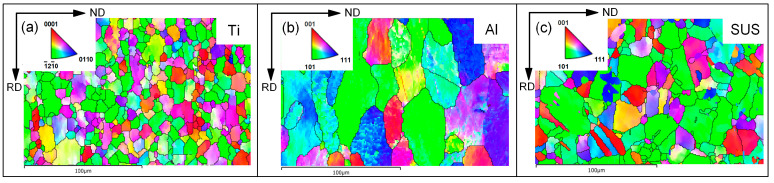
EBSD inverse pole figure (IPF) maps of constituent layers: (**a**) Al1050, (**b**) TA1, (**c**) SUS430.

SUS430/Al1050/TA1 laminated sheets used in this study were fabricated through cold rolling followed by annealing at 550 °C for 1 h, with final individual layer thicknesses of 0.5 mm, 1.2 mm, and 0.3 mm, respectively. Figure 1 presents the cross-sectional surface morphology of the LMC sample observed via scanning electron microscope (SEM), revealing flat and coherent interfaces without detectable microvoids. The element distribution lines measured using an equipped energy-dispersive spectroscopy (EDS) along the corresponding scanning line are extremely steep, indicating negligible intermetallic compound layers formation at the interfaces. These well bonded interfacial characteristics satisfy the fundamental assumptions of the developed crystal plasticity model. The initial crystallographic textures of each constituent metal obtained by electron backscatter diffraction (EBSD) are illustrated in Figure 2. The TA1 layer displays a uniform microstructure dominated by equiaxed grains. The Al1050 layer exhibits a typical rolling deformation texture, characterized by flattened and elongated grains along the rolling direction. The SUS430 layer features a bimodal grain structure comprising both coarse and fine grains. All EBSD measurements were conducted along the longitudinal cross-section using a 1 μm step size.

### 2.2. Mechanical Tests

**Figure 3:**
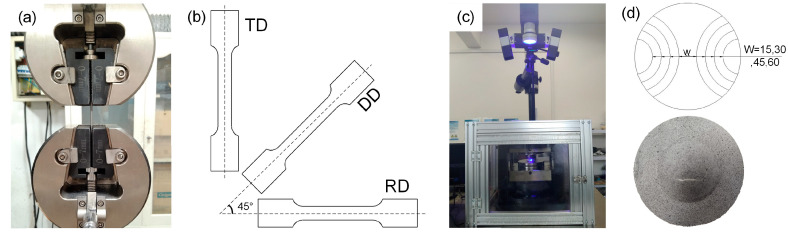
Experimental schematic diagram. (**a**) Uniaxial tension setup, (**b**) tensile specimens, (**c**) Nakazima test system, (**d**) specimen geometry and deformed shape of the Nakazima test.

Tensile properties were evaluated using an electronic universal testing machine, as shown in Figure 3a, at room temperature with a strain rate of 10^−3^·s^−1^. The geometric dimensions of the specimens were designed in accordance with the ASTM E8 standard, with dimensions of 25 × 6 mm (gauge length × width) [5]. Repeated uniaxial tensile tests were carried out oriented along the rolling direction (RD), diagonal direction (DD), and transverse direction (TD) to characterize the mechanical behavior of the LMC sheet. Figure 3b illustrates the three sampling directions.

Nakazima tests were performed to determine the FLD of the SUS430/Al1050/TA1 laminated sheets. The experimental punch stroke and load were recorded by a computer-aided control system connected to the hydraulic testing machine, while the deformation field was monitored using the ARAMIS^®^ system (GOM). The experimental setup for Nakazima tests is shown in Figure 3c. The circular specimens with a diameter of 90 mm were prepared into a series of five arc-shaped specimens with different widths, which increase from 15 mm to 90 mm, representing strain paths from uniaxial tension to equibiaxial tension. Figure 3d illustrates the required dimensions for the Nakazima samples. The specimens were wire cut along the major strain direction parallel to the rolling direction and then sprayed with black and white paint to create the speckle pattern on the surface, as exemplified by the equibiaxial tension deformed specimen shown in Figure 3d. The forming limit strains were obtained through a full-field digital image correlation (DIC) camera. In the hemispherical punch stretching test, unfavorable friction conditions between the punch and the specimen significantly restrict the plastic flow in the central contact region, leading to fracture initiation in the arc-shaped edges. To minimize the influence of friction on the determination of the FLD, lubrication conditions between the LMC sheet and the punch must be optimized during experimental procedures. In this study, a polyethylene sheet coated with vaseline was placed between the blank and the die to minimize friction. The specimen was deformed by a hemispherical punch of 40 mm in diameter descending at a constant speed of 0.5 mm·s^−1^. Each test was performed three times for each tested specimen to ensure reproducibility.

## 3. CPFE–MK Framework

### 3.1. Crystal Plasticity Theory

Crystal plasticity theory establishes a constitutive framework in which the total deformation can be decomposed into elastic and plastic parts. Upon application of external loads, both deformation modes are activated simultaneously. The kinematics of crystal plasticity are fundamentally described by the multiplicative decomposition of the deformation gradient, as follows:

(1) $$F=F^{e}F^{p}$$

where the elastic deformation gradient *F^e^* is caused by lattice distortions and rigid body rotation, and *F^p^* represents the non-recoverable plastic deformation, including specific slip system activation and twinning.

The crystal deformation velocity gradient tensor *L* in the current configuration is defined as:

(2) $$L=\dot{F}F^{-1}=L^{e}+L^{p}$$

where *L^e^* and *L^p^* can be regarded as the elastic and plastic parts associated with *L*. The type and number of slip systems typically vary depending on the crystal structure. The metal composite sheet investigated in this study contains the three most common metallic crystalline structures: Al (FCC), SUS430 (BCC), and TA1 (HCP). The plastic deformation of FCC crystals is predominantly governed by 12 distinct {111}<110> slip systems, which are realized by dislocation glide along {111} close-packed planes and in <110> directions. The BCC crystals exhibit more complicated slip mechanisms involving 24 potential slip systems, comprising 12 {110}<111> systems and 12 {112}<111> systems. The slip systems of TA1 include basal slip on {0001}<11-20>, <a> prismatic slip {10-10}<11-20>, <a> pyramidal slip {10-11}<11-20>, and <c+a> pyramidal slip systems encompassing both first-order {10-11}<11-23> and second-order {11-22}<11-23> configurations. The TA1 twinning mechanism can be classified according to the stress state into tensile twinning characterized by {10-12}<-1011> slip systems and compression twinning governed by {11-22}<11-2-3> slip systems. Twinning is a crucial deformation mechanism, particularly in HCP structural materials, and its kinetics significantly influence macroscopic mechanical behavior. The twinning mechanism can be treated as pseudo-slip and incorporated into a unified constitutive framework, together with conventional dislocation slip, to achieve multi-mode plastic deformation analysis. Thus, *L^p^* can be expressed as:

(3) $$L^{p}=\left(1-\sum_{\beta =1}^{N_{tw}}f^{\beta }\right)\sum_{\alpha =1}^{N_{s}}{\dot{\gamma }}^{\alpha }m^{\alpha }⊗n^{\alpha }+\sum_{\beta =1}^{N_{tw}}\gamma_{tw}{\dot{f}}^{\beta }m^{\beta }⊗n^{\beta }$$

The slip/twinning direction and the normal to the slip/twinning plane before crystal deformation are represented by the unit vectors *m^α^* and *n^α^*. *N_tw_* and *N_s_* are the total number of potentially active twinning and slip systems. The relative contributions of slip and twinning deformation mechanisms are assessed based on the twinning volume fraction *f^β^*.

(4) $$f^{\beta }=\frac{\gamma^{\beta }}{\gamma_{tw}}$$

where *γ^β^* is the cumulative shear strain of the twinning system and *γ_tw_* is a constant that represents the twinning shear. The shear strain rate ${\dot{\gamma }}^{\alpha }$ and ${\dot{\gamma }}^{\beta }$ of slip and twinning system are usually expressed in the form of a power function equation, as follows:

(5) $${\dot{\gamma }}^{i}={\dot{\gamma }}_{0}{\left[\frac{\tau^{i}}{g^{i}}\right]}^{\frac{1}{m}}\mathrm{sign}\left(\tau^{i}\right)\;\;\;\;\;\;i=\alpha /\beta$$

where ${\dot{\gamma }}_{0}$ is the reference shear strain rate, which is a constant related to the material; *τ^i^* is the resolved shear stress; *m* is the strain rate sensitivity; and *g^i^* is the critical resolved shear stress, which characterizes the degree of hardening in the slip system. During plastic deformation of metallic materials, work hardening occurs due to dislocation interactions between slip systems or the interaction between slip and twinning. This study employs a rate-dependent hardening model to characterize this behavior.

(6) $${\dot{g}}^{\alpha }=\sum_{j=1}^{N_{s}+N_{tw}}h_{ij}\left|{\dot{\gamma }}^{i}\right|$$

For slip and twinning systems, strain hardening is described by the evolution of *g^α^* through incremental relationships, where *h_ij_* is the hardening moduli.

(7) $$h_{ij}=qh(\gamma )$$

where *q* is the ratio between the experimentally determined self and latent hardening coefficient, typically ranging from 1 to 1.4. Peirce and Asaro et al. employed a simplified formulation to characterize the self-hardening modulus [28].

(8) $$h(\gamma )=h_{0}{\mathrm{sech}}^{2}\left|\frac{h_{0}\gamma }{\tau_{s}-\tau_{0}}\right|$$

where *h*_0_ is the initial hardening modulus, *τ*_0_ is the initial critical shear stress, *τ_s_* is the shear stress in the initial stage, and *γ* is the Taylor cumulative shear strain on all slip and twinning systems. The crystal plasticity theory was applied to Abaqus/Standard through a UMAT user subroutine.

### 3.2. RVE Generation

The predictive accuracy of the CPFE model is highly dependent on the accurate characterization of the polycrystalline material. Based on the initial crystallographic texture data obtained from EBSD, a realistic 3D RVE of the measured microstructure was constructed. This study developed a MATLAB algorithm to process crystallographic orientation data. By editing the INP file, a virtually reconstructed CPFE model was implemented in ABAQUS (version 2021), integrating three key functions: (i) assigning crystal plasticity constitutive parameters to each grain, (ii) mapping crystal orientation onto grains using Euler angles, and (iii) determining the element–grain assignment relationship based on the specified number of grains. A displacement-based periodic boundary condition (PBC) was applied to establish displacement compatibility between opposing surfaces of the RVE. This approach is valid as the microstructure is characterized by a relatively uniform distribution of grain sizes and orientations. The application of PBCs enables the RVE model to approximate a larger part of the material, ensuring the RVE behaves as a physically continuous domain. This method not only satisfies the minimum size requirement for the RVE but also ensures high-accuracy computational results within the limited deformation range [29]. The laminated structure maintains periodicity perpendicular to the thickness direction throughout the loading process. The four surfaces subject to boundary conditions in the RVE are represented by Front, Back, Left, and Right, with front-back and left-right nodal pairs requiring strict correspondence. The displacement relationship can be described as follows:

(9) $$u_{i}^{Front}=u_{i}^{Back}+\varepsilon \times u_{i}(x)$$

(10) $$u_{i}^{Right}=u_{i}^{Left}+\varepsilon \times u_{i}(y)$$

Nodes on the positive and negative X-surfaces are labeled as *u^Front^* and *u^Back^*, respectively. *u_i_*(*x*) and *u_i_*(*y*) represent the displacement at corresponding nodes in the RVE, ε is the applied strain tensor, and *εu_i_*(*x*) denotes the displacement vector between corresponding nodes located on opposing boundary surface pairs induced by equivalent strain. Nodes on Y-surfaces are defined in the same way. In the modeling process, the periodic boundary conditions were first implemented by modifying the .inp file to add “EQUATION” in the Interaction module of ABAQUS, which provides a time-efficient solution for models containing numerous nodes.

### 3.3. M–K Model

**Figure 4:**
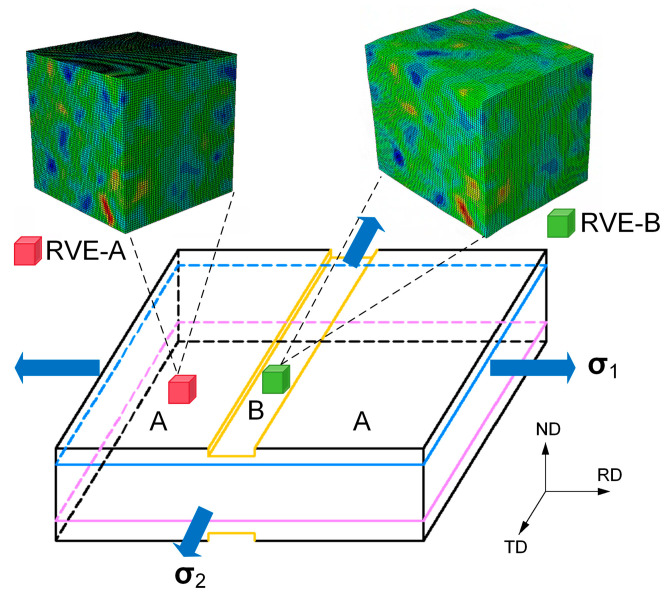
Schematic of the combined M–K method and CPFE model in the multi-scale framework.

The M–K model was combined with the CPFE method to analyze the forming limits of laminated sheets. The fundamental assumption of the M–K model is based on the existence of initial surface defects, which account for the unavoidable thickness inhomogeneity in sheet materials. This geometric imperfection is represented by a groove configuration, where region B corresponds to the groove region and region A designates the uniform region. Therefore, the polycrystal model requires the establishment of two RVEs with identical initial textures, one representing the narrow band region and the other representing the region outside the band, as illustrated in Figure 4.

M–K model assumes that the force equilibrium requirement along the major strain direction is satisfied, namely $\sigma_{1}^{A}t^{A}=\sigma_{1}^{B}t^{B}$, and that the geometric compatibility condition $d\varepsilon_{2}^{A}=d\varepsilon_{2}^{B}$ is met, where the strain increment $d\varepsilon_{2}^{A}$ in homogeneous region A is the same as that in the imperfection region B $d\varepsilon_{2}^{B}$. The subscripts 1 and 2 represent the major strain direction and the minor strain direction. The initial imperfection is assumed to be perpendicular to the direction of the major stress, which is characterized by the inhomogeneity factor $f_{0}=\frac{t^{B}}{t^{A}}$, where *t* denotes the sheet thickness and the subscript ‘0’ denotes the initial state. The strain along the thickness direction can be expressed according to the conventional definition of logarithmic strain as: $t=t_{0}\exp(\varepsilon_{3})$. Then, the imperfection factor during the deformation process can be obtained: $f=f_{0}\exp\left(\varepsilon_{3}^{B}-\varepsilon_{3}^{A}\right)$. The strain path is defined by a constant strain rate ratio $\rho =\frac{d\varepsilon_{2}}{d\varepsilon_{1}}$. By combining the above core assumptions and defining the equilibrium, equations between region A and region B can be written as:

(11) $$\sigma_{1}^{A}\exp\left(\varepsilon_{1}^{A}-\varepsilon_{1}^{B}\right)=f\sigma_{1}^{B}$$

**Figure 5:**
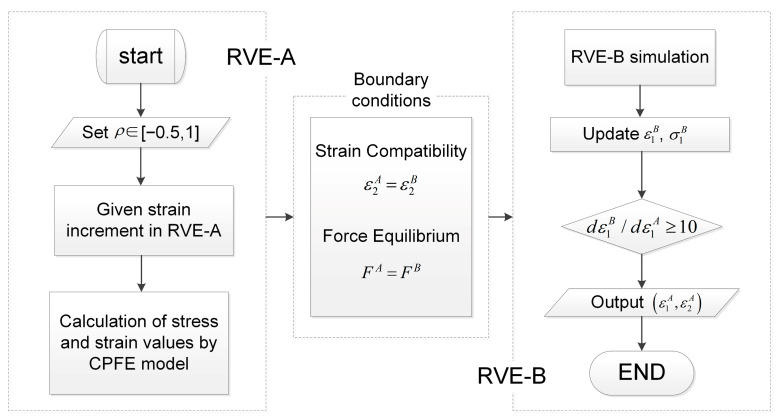
Flowchart of the CPFE–MK method.

Assuming that the strains in the safe area are proportional and that compatibility and equilibrium conditions are satisfied across the imperfection band. Based on combining the above M–K basic equations, the strain increment in the imperfection region is then greater than that in the homogeneous zone. M–K necking condition can be defined in various ways. The flow localization is considered to occur when the strain increment ratio exceeds a critical value: $d\varepsilon_{1}^{B}>10d\varepsilon_{1}^{A}$. At this stage, the computation is stopped and the calculated corresponding limit strains are reached. The overall flowchart for predicting the FLD through the CPFE–MK method is shown in Figure 5. The process began by simulating RVE-A, which represented the uniform region, under a preset constant strain rate ratio *ρ*. Subsequently, the simulation data from RVE-A were processed based on the assumptions of force equilibrium and strain compatibility, which form the core equations of the M–K model. The necessary boundary conditions required for RVE-B representing the imperfection region were derived. Finally, RVE-B was simulated under these calculated boundary conditions until the instability criterion was met.

## 4. Results

### 4.1. RVE Size Determination

**Figure 6:**
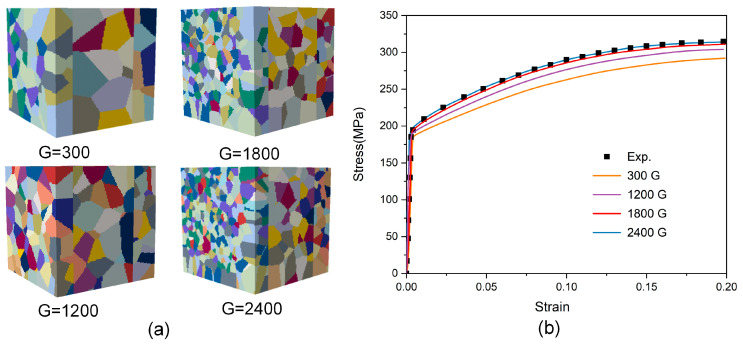
(**a**) Generated RVE of different number of grains. (**b**) Comparison of numerical and experimental stress–strain curves.

**Figure 7:**
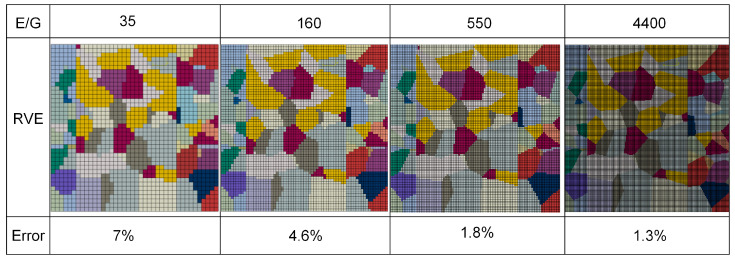
Determining the average number of E/G used in the CPFE simulation through error analysis.

In establishing the RVE model, the primary consideration is to define the dimensional parameters, specifically the grain count within the RVE and element assignment per grain. The maximum grain count is constrained by microstructural morphological heterogeneity and the mesh resolution. Nevertheless, the RVE must contain a sufficient number of grains to accurately capture microstructural feature evolution during deformation. In addition, an excessive grain count would result in unreasonable or even unaffordable computational costs, even though it would make the response of the RVE model statistically converge to the macroscopic behavior of real materials. To obtain the minimum grain count threshold, CPFE simulations via the RVE model require several iterations of calculations. The constitutive parameters of each material have been determined separately through tensile curves according to our previous studies [30]. The parameters used in the simulation were iteratively determined with the trial-and-error approach, as listed in Table 1. The corresponding stress–strain curves for the models containing 300, 1200, 1800, and 2400 grains are shown in Figure 6. RVE models with fewer grains exhibit larger grain sizes, resulting in lower predicted flow stress and higher dispersion of the corresponding stress–strain curves. When the number of grains reaches 1800, it can be considered that the experimental curve is in agreement with the simulation curve. For grain counts exceeding 1800, the model can be used to represent a polycrystalline aggregate, indicating that the RVE with a sufficient number of grains can reduce the influence of microstructural differences caused by modeling on CPFE simulation results. The number of elements required per grain (E/G) is also discussed. When the relative size of the E/G is lower, significant differences exist among the grain orientation distributions selected in the FE simulation, resulting in increased simulation errors. As the mesh density increases, the grain boundaries gradually become smooth. Meanwhile, the model with smooth grain boundaries is closer to the actual grain shape, giving simulation results with higher accuracy. Figure 7 shows the error values of tensile strength for the cases of 35, 160, 550, and 4400 elements per grain (in average sense). It is found that the prediction of the model can be satisfied by dividing each grain into 550 elements.

### 4.2. FLD Analysis

**Figure 8:**
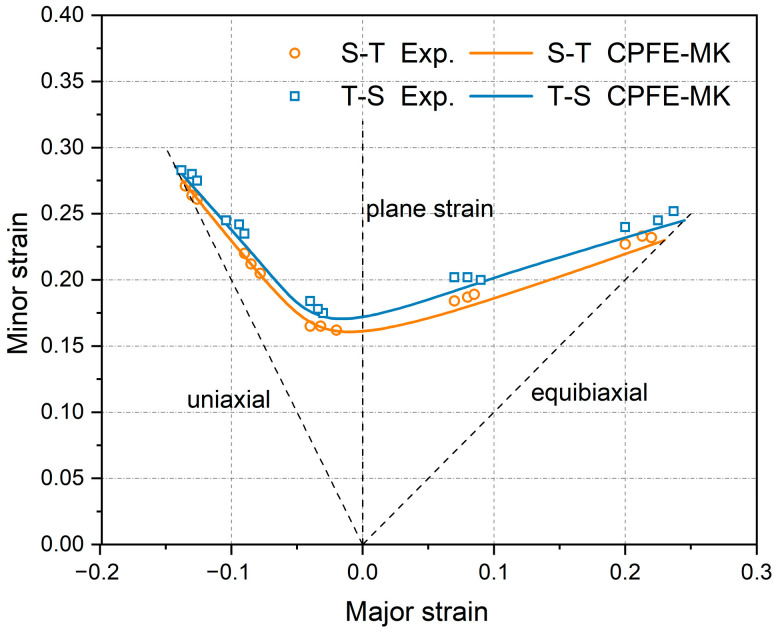
FLD prediction of T-S and S-T stacking sequences compared with experimental results.

The formability of SUS430/Al1050/TA1 laminates was further assessed using the established CPFE–MK model. The RVEs incorporating realistic texture characteristics were employed to simulate various loading paths. The FLD of the multi-layer structure was predicted using the M–K theory as the criterion for determining the forming limits. The limit strain under the plane strain stretching condition was chosen for iterative fitting to obtain the inhomogeneity factor (*f*_0_) of 0.995. Given the asymmetric structure of the laminated sheets, T-S and S-T lay-ups were considered, corresponding to cases where the steel layer or the Ti layer was in contact with the die, respectively. The predicted necking strains considering both stacking sequences based on the aforementioned method were compared with the experimental values as presented in Figure 8. The modified CPFE–MK method demonstrates reasonable effectiveness for predicting the mechanical performance of multi-layer structures. To assess the accuracy of FLD prediction, the average error values of the major strain and minor strain are calculated using Equation (12). N is the total number of specimens; $\varepsilon_{i}^{\mathrm{Exp}}$ and $\varepsilon_{i}^{\mathrm{CPFE}}$ are the experimental and CPFE–MK predicted limit strains. Table 2 presents the errors in major and minor forming limit strains between numerical simulations and experimental measurements.

(12) $$\mathrm{Error}=\frac{1}{N}\sum_{i=1}^{N}\left|\frac{\varepsilon_{i}^{\mathrm{Exp}}-\varepsilon_{i}^{\mathrm{CPFE}}}{\varepsilon_{i}^{Exp}}\right|\times 100$$

According to the results of the above table, the simulated points on the left side of the FLD are consistent with the experimental results, while the simulated values for the right-side region are slightly higher than the experimentally observed values. Even with such a minor inconsistency, the validity of the prediction model is verified. The crystal plasticity model can effectively predict the forming limits of multi-layer LMC sheets containing FCC, BCC, and HCP structures under various strain paths during the plastic forming process, with high accuracy of the FLD.

## 5. Discussion

**Figure 9:**
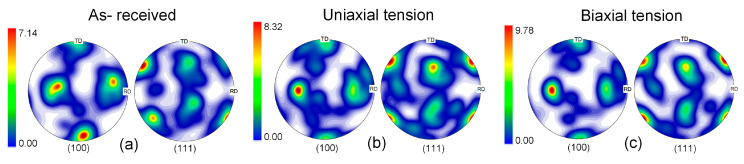
(100) and (111) pole figures of the Al1050 layer: (**a**) initial texture in the as-received condition; simulated deformed texture after (**b**) uniaxial tension and (**c**) biaxial tensile tension.

**Figure 10:**
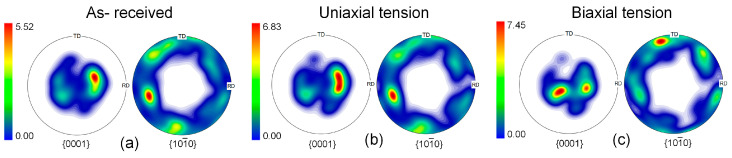
{0001} and {10$\bar{1}$0} pole figures of the TA1 layer: (**a**) initial texture in the as-received condition; simulated deformed texture after (**b**) uniaxial tension and (**c**) biaxial tensile tension.

Numerical simulations and experimental measurements indicate that the SUS430/Al1050/TA1 LMC sheet exhibits good formability. It is worth noting that the formability of polycrystalline materials demonstrates high sensitivity to texture characteristics [31,32]. The initial texture of each layer was characterized by processing EBSD data. The pole figures of the investigated Al layer and Ti layer are, respectively, displayed in Figure 9a and Figure 10a. The Al1050 layer features a moderate Goss texture, whereas the TA1 layer shows a distinct basal texture with the basal pole tilted toward the transverse direction. The crystal orientation data of the constituent layers before instability under uniaxial tension (*ρ* = −0.5) and biaxial tensile tension loading paths (*ρ* = 1) were calculated using a Python (version 3.7) script. The deformed textures after 15% tension generated from the simulation were processed and analyzed using the MTEX Toolbox in MATLAB 2016. It is worth noting that texture evolution is primarily governed by the strain path and the accumulated plastic strain. For a given *ρ* value, the macroscopic loading conditions imposed on the RVE are identical for both the T-S and S-T lay-ups. Although the stacking sequence differs, it does not lead to a significant difference in the deformation mode. As a result, the texture evolution trends of each constituent layer are similar in both cases. As shown in Figure 9b,c, texture evolution does occur during deformation, though the extent of this evolution is relatively moderate. In the ductile A1050 layer, limited texture evolution is observed under a 15% strain condition, not as pronounced as under severe deformation, and does not involve distinct reorientation. Pole figure analysis reveals that an enhancement of the Goss texture can be observed by comparing the initial texture with the deformed texture of the Al layer, with the maximum pole density in the pole figures increasing from 7.14 in the initial material to 9.78. Similarly, for the TA1 of the HCP structure, a similar phenomenon was observed. As displayed in Figure 10b,c, both basal slip systems and non-basal slip systems in the HCP structure are active during different deformation processes. Deformation causes partial basal rotation while reducing basal pole intensity. Especially under biaxial stretching conditions, no obvious basal texture is observed, as shown in Figure 10c. The hardening effect caused by basal slip results in uniform deformation of the sheet, contributing to improved formability. This indicates that relatively uniform plastic deformation did not induce a significant change in grain orientation. The formation of new orientations requires higher strain or more complex deformation modes.

**Figure 11:**
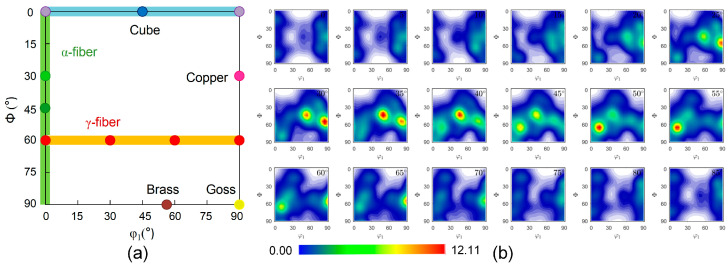
(**a**) Locations of typical orientations and fiber texture at φ_2_ = 45° section. (**b**) The examined ODFs of the steel layer.

**Figure 12:**
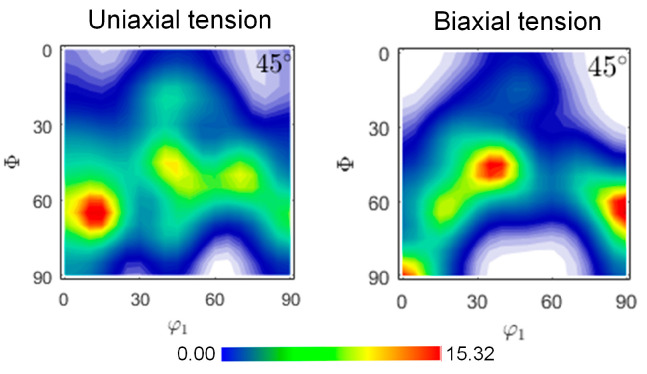
Simulated deformed textures of SUS430 layer.

The orientation distribution function (ODF) sections were estimated for the SUS430 layer in comparison with the ideal orientations to characterize the texture, as presented in Figure 11. The textures were characterized using ODF sections at constant φ_2_ intervals with a 5° half-width. It reveals that the steel layer exhibits a strong γ-fiber texture with the orientation density distributed. Textures obtained at limit strains for uniaxial tension and biaxial tensile tension loading paths are presented in Figure 12. From the ODF sections of the steel layer after deformation with φ_2_ = 45°, it can be seen that deformation has induced significant texture evolution along the γ-fiber. Uniaxial tension promotes the intensity of the grains oriented toward φ_1_ = 0°. During equibiaxial tension, the texture changes are more pronounced, which strengthens the φ_1_ = 30° and 90°orientations. T-S lay-up situation exhibits relatively higher forming performance from the observation of the FLD. The γ-fiber texture of the steel layer promotes formability, while the basal texture of the TA1 layer has a suppressive effect [32,33,34,35,36]. The CPFE model successfully reproduces these microstructure-property connections and provides an efficient and accurate predictive tool for multi-layer LMC structures.

## 6. Conclusions

The present study established a multi-scale framework, integrating the crystal plasticity finite element model with M–K theory to predict the FLDs for SUS430/Al1050/TA1 laminated sheets. The advantages of the efficiency prediction capability of the macro-model and the accurate mechanistic representation of the micro-model have been combined. The CPFE–MK methodology was further extended to the application of multi-layer LMCs, considering the crystallographic structure of each constituent material. The following conclusions can be drawn:

1. The size effect on prediction accuracy was investigated through convergence analysis. The grain count and mesh density of the RVE were calibrated by fitting the simulated stress–strain curves against uniaxial tensile data. The optimal size RVE predictions were in good agreement with the tensile results.
2. The modified CPFE–MK model is reasonably effective in predicting the FLDs for the SUS430/Al1050/TA1 sheet under two stacking sequences, namely T-S and S-T lay-ups. The predictions were in high agreement, with average absolute errors of less than 6% and 5% for the T-S and S-T lay-ups, respectively, when validated against the Nakazima test results.
3. The effects of both initial and deformation-induced textures on the formability of the LMC sheet were analyzed. The beneficial γ-fiber texture in the SUS layer and the detrimental basal texture in the TA1 layer microscopically explain the superior performance of the T-S lay-up over the S-T lay-up. CPFE–MK simulations under various strain paths also revealed enhanced γ-fiber texture and weakened basal texture after deformation.

## Figures and Tables

**Table 1:** Parameters determined for CPFEM.

Material	${\dot{\gamma }}_{0}$ (s^−1^)	*h*_0_ (MPa)	*τ*_0_(MPa)	*τ_s_* (MPa)	*m*
SUS430	1	110	81	122	0.05
Al1050	1	63	49	83	0.05
TA1 Bas<a>	0.001	19	188	230	0.05
TA1 Pri<a>	0.001	37	74	92	0.05
TA1 Py<a>	1	320	695	884	0.05
TA1 Py_1_<c + a>	1	320	622	807	0.05
TA1 Py_2_<c + a>	0.001	18.8	630	788	0.05
Tw (ten.)	1	245	184	296	0.05
Tw (com.)	1	245	358	496	0.05

**Table 2:** Numerical-experimental errors in forming limit strains.

		T-S Error	S-T Error
Sample1	Major strain	2.8%	1.5%
	Minor strain	3.4%	3.1%
Sample2	Major strain	2.7%	0.4%
	Minor strain	3.2%	0.3%
Sample3	Major strain	1.8%	1.6%
	Minor strain	5.3%	2.2%
Sample4	Major strain	3.1%	4%
	Minor strain	5.2%	4.6%
Sample5	Major strain	2.9%	3.7%
	Minor strain	5.7%	4.8%

## Data Availability

The original contributions presented in this study are included in the article. Further inquiries can be directed to the corresponding author.
